# Development of a tool to identify barriers and enablers to practice innovation in midwifery: A participatory action research study

**DOI:** 10.18332/ejm/157459

**Published:** 2023-01-30

**Authors:** Sara D. Davis, Sara Bayes, Sadie Geraghty

**Affiliations:** 1 School of Nursing and Midwifery, Edith Cowan University, Perth, Australia; 2 School of Nursing, Midwifery and Paramedicine, Australian Catholic University, Melbourne, Australia; 3 School of Nursing, Midwifery, Health Sciences and Physiotherapy, University of Notre Dame Australia, Fremantle, Australia

**Keywords:** readiness for change, implementation, midwifery, tool development, change management

## Abstract

**INTRODUCTION:**

Transferring research evidence into midwifery practice is fraught with challenges and obstacles. Implementation tools can streamline the process and are most effective when they are discipline-specific; however, there are currently no midwifery specific implementation tools. The aim of this study was to develop a midwifery specific tool to identify barriers and enablers to evidence-informed practice change within the clinical setting.

**METHODS:**

Participatory action research methodology was employed to ensure potential end-users contributed to content and format of the tool. Purposeful sampling ensured participants were selected from a range of midwifery practice settings in Western Australia and the United Kingdom. Data were collected through stakeholder advisory groups (SAGs) and online surveys.

**RESULTS:**

Ten midwives participated in this project. Consultation occurred through face-to-face SAG meetings and online surveys until consensus was reached among participants about the content, format, and functionality of the end product which we called the ‘Midwifery Tool for Change’ (MT4C).

**CONCLUSIONS:**

To our knowledge, the MT4C is the first readiness for change context assessment tool specific to midwifery practice settings. Evaluation of the MT4C in real-world practice change implementation initiatives will enable further refinement of the tool.

## INTRODUCTION

Healthcare professionals, including midwives, are expected to utilize research findings in their day-to-day practice to ensure best-evidence-based and optimized outcomes for women and babies^1,2^. This mandate recognizes that quality healthcare is a combination of desired outcomes and research evidence, and that ‘the call for evidence-based quality improvement and healthcare transformation underscores the need for redesigning care that is effective, safe and efficient’^3^. However, it is also acknowledged that there exists a distinct discrepancy between what should be done and what is actually done in terms of evidence-based care^4-7^.

Implementation science looks to establish methods to bridge the gap between knowledge and practice, which is both time-consuming and multifaceted^8^. Traditionally, research findings are used to develop clinical guidelines and policies; however, providing evidence to clinicians in the form of guidelines and policy alone does not automatically change practice^4^. In a reflection on the process of implementation of evidence into midwifery care, Hunter^4^ likens the gap between dissemination and implementation of research findings to a ‘black box’ in that ‘it is a complex process whose internal workings are unclear and at times puzzling’. Hunter goes on to suggest the contents of this black box contain elements that will present various barriers to the midwife looking to implement change, which include the practice context, the issue, and knowledge users. These elements are similarly recognized, to a greater or lesser degree, as being impactful in healthcare settings more broadly, and feature in many other implementation science theories and frameworks such as the Consolidated Framework for Implementation Research (CFIR)^9^ and the Promoting Action on Research Implementation in Health Services (PARIHS) model^6,10^.

Although implementation tools are used by midwives^11-16^, they have been developed with other professions and professional settings in mind such as nursing; there are no midwifery specific evidence-based change implementation tools available to capture the factors that impact the success of practice change initiatives in the midwifery context. Change leader midwives have identified barriers to practice change such as: cynicism and mistrust of proposed changes by staff throughout the multi-disciplinary team; a lack of understanding amongst doctors resulting in a resistance to midwifery driven change; and lack of management support and funding issues^17^. Central to the drive for the development of midwifery specific implementation aids is the fact that midwifery is a stand-alone profession and as such is fundamentally different to nursing. Historically within the UK and latterly elsewhere within the Commonwealth, the Midwives Act of 1902 heralded the recognition of Midwifery as a profession in terms of ensuring public safety through formal education and registration of midwives. In Australia, the Nursing and Midwifery Board identify nursing and midwifery as separate professions through the provision of separate codes of conduct and standards for the practice for each^18-21^. A review of these documents demonstrates the subtle yet significance differences between each profession. Key aspects to the role of the midwife have been identified as the provision of skilled and collaborative care that is evidence-based and woman-centered, that is to say sympathetic to the unique requirements and experiences of the woman for whom the midwife is caring^22^. Midwives are autonomous practitioners, responsible for the holistic care of women throughout the continuum of pregnancy, labor, and birth. As such, midwives practice from a position of health promotion and supportive care through what is deemed to be a normal physiological process^22-24^.

The aim of this study was to develop a midwifery specific tool with which midwives can identify potential barriers and enablers to change in the clinical setting. This study builds on previous work conducted to determine the ‘fit’ for midwifery of the UK National Health Service (now defunct) Institute for Innovation and Improvement's (UK NHS III) ‘Spread and Adoption’ tool which was identified as potentially valuable but ‘not quite right’^25^. Bayes et al.^25^ found the tool was not appropriate for midwifery practice settings due to the lack of midwifery specific language and terminology, while access to the tool was also challenging for midwives in remote and rural areas of Australia where internet access is unreliable.

## METHODS

The Midwifery Tool for Change (MT4C) was developed using participatory action research (PAR) methodology to enable end-user representatives to contribute to its inception. In its simplest terms, PAR involves all people affected by a particular problem identifying or agreeing on the specific nature of that problem, deciding what can be done to resolve the issue and then carrying out the agreed action, evaluating how successful they were, and then trying again if necessary, using the lessons learned in the first attempt^26^.

The action research cycle model used for the development of the MT4C follows a four-stage process^27^. In this model, each cycle involves four temporally ordered stages through which participants' vision for the outcome of the process is captured (Stage 1); an action plan is developed (Stage 2); implementation of the action plan occurs, and the ‘product’ of the process is created (Stage 3); and the product is then reviewed (Stage 4)^27^. The research team comprised midwifery academics with many years of clinical experience. The present study forms part of the first author's PhD research and arose from the view of a practicing midwife attempting to affect meaningful change within the clinical setting ensuring that practice is not only evidence-based but also woman-centered. This driving force to affect a change represents stage one of the action research cycle. Stage two was the creation of the draft MT4C. This was done using the feedback obtained from participants in the Bayes et al.^25^ study. Stage three occurred through the collection of feedback at stakeholder advisory group (SAG) meetings and via online surveys. Stage four is currently underway with the MT4C being evaluated by change leader midwives in Australia and the UK through application to actual change implementation activities.

Recruitment of participants primarily occurred in WA to facilitate the SAG meetings. The research team felt that having face-to-face SAG meetings would be more effective at the early stages of the study. Focus groups provide a valuable form of data collection with the PAR methodology as they enable participants to collectively ‘generate meaningful opinions, suggestions and feedback’ enabling researchers to ‘[listen] to the perspective of key stakeholders and [learn] from their experiences of the phenomenon … tilting the balance of power toward the group’^28^. UK participants were recruited to evaluate if the challenges experienced by midwives were broadly similar across both countries. Purposive sampling was used and participants were required to be practicing midwives who had previously been involved in a change implementation activity with experience of leading practice change initiatives. The study was publicized via the Edith Cowan University social media platform and by The Australian College of Midwives WA branch in the form of an e-bulletin to membership. A total of 14 change leader midwives from a range of clinical settings consented and participated in the study across the two rounds of consultation (Table 1). Although no participants requested to withdraw from the study, 4 participants who accessed the online survey did not comment or make recommendations leaving a total of 10 active participants.

**Table 1:** Demographics of participants

*Primary area of practice*	*WA participants (n=11)*	*UK participants (n=3)*
Tertiary public hospital	5	1
Publicly funded homebirth program	1	
Regional hospital	1	1
Private hospital	3	
Midwife in private practice	1	1

Two forms of data were collected between September and October 2018: two rounds of focus group discussions/SAG meetings (six participants), and two online surveys created using Qualtrics (eight participants). Table 1 shows participants' primary area of practice which was the only form of demographic data collected. For the first round of data collection, SAG meeting and online survey participants were presented with an initial draft of the proposed MT4C and asked to consider its ‘fit’ for the midwifery context. This first draft comprised 24 questions (elements) developed from the UK NHS III Spread and Adoption tool that had been modified to take account of the suggestions resulting from earlier research by Bayes et al.^25^. The 24 elements were grouped into three domains: People, Innovation and Context. These domains were kept due to their simplicity and the desire for the MT4C to be concise.

Each element was addressed and evaluated in turn for midwifery practice fit. Participants were invited to make other suggestions for the tool including changes to its functionality or format. Participants were also asked to reflect upon their previous implementation activities and identify whether the barriers and enablers they encountered would have been identified by the proposed tool.

The SAG meetings took place at a central location in Perth, Western Australia, chosen for ease of access for participants. The first SAG meeting was facilitated by the first author with the second author assisting and making field notes. The second SAG was facilitated by the first author alone. Both meetings lasted approximately two hours and were audio-recorded, transcribed and collated with the data from the online survey. The transcription of the first SAG meeting was combined with the results of the online surveys and re-read several times ensuring familiarization with the raw data. The changes recommended by participants related to the language, content and structure of the tool itself, personnel, and processes of change. The recommended changes identified in round one were applied creating a second draft of the MT4C, which consisted of 18 elements in the same three domains as version one and formed the basis of the second SAG and online survey. The same data analysis process used in the first round of data collection was employed, with no new changes or recommendations identified and the tool was finalized. The trustworthiness of the findings was further affirmed through independent analysis of a sample of the data by the first two authors.

## RESULTS

During the first SAG meeting, it became apparent that some participants were not familiar with Implementation Science or the concepts of frameworks to assist change implementation activities. There was also uncertainty amongst participants about how the proposed tool would be used. This was an unexpected finding, although, upon reflection, the lack of midwifery specific implementation frameworks should have prepared the researchers for this eventuality. The discovery of a potential lack of awareness and understanding amongst midwives led to the development of an explanatory video which was forwarded to participants to ensure the purpose and intention of the study was clear and to facilitate stage three of the PAR cycle.

At the first SAG meeting, the notion of culture was discussed at length. The original UK NHS III Spread and Adoption tool contains an element aimed at identifying whether the organizational culture is appropriate for change to occur. Participants agreed that culture is crucial to an organization's ability to embrace and successfully achieve growth or not and is potentially a ‘deal breaker’ in terms of successful implementation, as was identified:

*‘It's [ensuring an organizational culture of respect trust and open communication] almost like an entire side project to that thing you're actually going to do.’* (Participant 3)

*‘If you did identify it [organizational culture] as a barrier, what do you then do? If you are biting off a massive thing to try and address that barrier ... it might be a rabbit hole you never come out of.’* (Participant 1)

This feedback resulted in the removal of the element and the addition of a note in the tool's preamble about the importance of ensuring a culture of respect and open communication within the organization as an underpinning of successful change implementation.

Participants from both UK and WA made recommendations regarding the separation of clinicians within the tool. The original UK NHS III Spread and Adoption tool refers to nurses and doctors within the same statement. For the first draft of the MT4C, any reference to ‘nurses’ was directly replaced with ‘midwives’, however, to identify the potential barriers or enablers to change within a midwifery setting, it was suggested that within the tool we:

*‘… separate active commitment of change leaders into two questions: doctors and midwives.’* (Participant 4)

This recommendation was made by other participants who felt that midwives and doctors needed to be separated as the approaches and attitudes of each discipline can differ significantly regarding potential changes in practice. It was observed:

*‘… the midwives might be the barrier, or the doctors might be the barrier, or it could be both.’* (Participant 2)

Although there was concern with the potential powerlessness of midwives recounting:

*‘In my experience nothing much changes unless the doctors are behind it … Midwives as a sole profession are pretty powerless.’* (Participant 3)

It was also keenly expressed that the tool should empower midwives:

*‘… let's not give away power, I think there is already a lot ... in our guidelines for consultation and referral and our guidelines for practice ... I'm not interested in giving away power on a local midwifery thing ... they don't ask us about surgical.’* (Participant 1)

Within the SAG there was also a lengthy discussion regarding the notion of ‘Active midwifery management and clinical leadership’ factors that had appeared together in the first element of the initial draft of the MT4C. Participants explored the implicit meaning behind these terms with the observation:

*‘To me it implies that you either have an active and motivated and you know, willing group, rather than an inactive stagnant one.’* (Participant 1)

Similarly, the idea to address midwifery managers and leaders in two separate elements was made by participants as these two roles may be held by different personnel; for example, a midwifery manager may not be a leader and vice versa. As was observed via the online survey:

*‘[the Element] seems to ask two questions in one question, Midwifery management is one aspect, midwifery leadership another.’* (Participant 4)

Although at the SAG, the merit was questioned in making such a distinction between midwifery managers and leaders:

*‘... you kind of can't really split them because you can have managers who are not clinical, but do they have the same experience as the clinical leadership? You need to have both for change.’* (Participant 1)

At the end of the first round of consultation, the decision was made to separate midwifery managers and midwifery leadership into two elements, and they remained in separate elements at the end of round two.

Participants stated that the tool needed to be simple, concise, and user-friendly; this resulted in five elements that were agreed to be repetitious being removed from the first draft of the MT4C (Supplementary file). Changes were also made to language and terminology making the tool more straightforward to use. This was concisely expressed on the basis that:

*‘… the target population to use this tool is midwives so I think if you use terminology that resonates with them ... it makes sense.’* (Participant 2)

Participants were also cognizant that the tool needed to address the fundamental aspects of successful change implementation so that all midwives regardless of experience can become drivers of change with one participant asserting that the MT4C could serve as a guide for young midwives with little experience of change implementation who:

*‘... may not think of the bigger organization influence [and needed] the prompts within the questions [to ensure] they have considered the impacts/barriers/need to source assistance.’* (Participant 9)

A more midwifery-centric approach is also reflected by the insistence of participants that women and babies were directly referenced within the tool. It was proposed that the language reflects this suggesting the phrase:

*‘This change will improve the experience of women and families birthing within the service.’* (Participant 7)

The involvement of women within the context of change implementation was deemed vitally important within the midwifery context, as was stated when the inclusion of an element addressing consumer support of the proposed change was discussed:

*‘That's a good question [to include]. Is this research … woman-centered because that's one of the central tenants of midwifery, isn' it? Is this improvement woman-centered?’* (Participant 10)

The inclusion of elements aimed at ensuring consumer involvement was further justified by comments which referred to the inclusion women as part of the multidisciplinary team involved with the successful implementation of change within a midwifery setting:

*‘It's one of our competencies and domains, it's one of the national safety standards, partnering with consumers ...’* (Participant 9)

The way users evaluate their organization's readiness for change was also discussed. Participants felt that the 5-point Likert scale used in the original UK NHS III Spread and Adoption tool was too broad and there was a universal dislike for the wording on these Likert scale elements. Instead, participants opted for a three-option selection using the terms: ‘We're there’, ‘We're nearly there’ and ‘We're nowhere near there’. One participant said:

*‘I would engage with that more than “agree”, “strongly agree” ... it's more humanistic’*. (Participant 3)

The three-response option was proposed by participants to be framed as a ‘traffic light for change’ system whereby green represented ‘We're there’, amber indicated ‘We're nearly there’, and red stood for ‘We're nowhere near there’. This format was endorsed in the second round of consultation with the comment that:

*‘The implication of the colors is very clear. It's very universal.’* (Participant 10)

The group further proposed that if an MT4C user selected a red or amber response, the requirement to note why before being able to move to the next element would be extremely helpful for reflection and discussion, and for comparison to future assessments:

*‘… if they score it as a red they are prompted to not go any further without saying why that's scored as a red … to make them really think about well why is that a red?’* (Participant 2)

*‘I think when you have lots of red, putting a comment in when you take it to your manager, it shows you have really put some thought into it and you've identified things that are barriers and can be changed.’* (Participant 1)

In the second consultation round, further formatting changes were made with the addition of a ‘go back’ function to allow users to review and amendment their responses. An element that considered the commitment of midwifery leadership to the change was also removed during the second round having been deemed repetitious.

A lengthy discussion regarding dissemination of the proposed change occurred at the second round SAG. Nowhere within the MT4C were users specifically asked whether the proposed change had been disseminated to the broader maternity care team. After re-reading each element and considering responses to each from the perspective of a midwife who had discussed the change with other stakeholders compared with one who had not, it was felt dissemination was implied through the wording of the elements:

*‘… you would have already ascertained whether the evidence was credible or not credible and you would have already had the tearoom discussion and then you would have gone, you know what, I am going to do something about that ...’* (Participant 2)

Furthermore, participants expressed the view that if the user had not disseminated the proposed change to the midwifery managers, there would be no way of knowing whether they were in support of the proposed change. This would, therefore, trigger an ‘amber’ or ‘red’ response to element one and the midwife would be required to add an explanatory note, which would indicate that the change needed to be discussed and disseminated.

In terms of the value of the tool to midwives at all levels, the group evaluated the MT4C positively:

*‘If you've got a good [Clinical Midwife] that comes up with this good idea or even a Level 1 person who comes up and just kinda goes, “what about [doing] this?”, you've got to have the supportive vehicle up the line (so) ... [the MT4C would mean] even a Grad can bring about change.’* (Participant 1)

In Australia, a ‘Clinical Midwife’ is a midwife who has greater responsibility than others; she/he may, for example, be a shift team leader. A ‘Level 1’ midwife in Australia is a midwife who works under the direction of a Clinical Midwife, a ‘Grad’ is a Midwife in their graduate year, i.e. in their first year of practice.

Participants were keen to ensure that the flow of elements within the first domain of ‘People’ placed the needs and wishes of women at the heart of the implementation process:

*‘… that's one of the domains of our practice, the recognition of the power imbalance in relationships and its influence on the woman ...’* (Participant 1)

This resulted in the re-ordering of elements putting the needs of women and babies at the beginning of this domain.

It was proposed by the research team, based on the findings of Bayes et al.^9^, that the MT4C be available via a desktop computer or a mobile hand-held device, and the participants made no additional suggestions with regard to the accessibility of the final MT4C. By providing the MT4C in an electronic application-based format, it was agreed that it will transcend the technological challenges of poor internet connections in rural and remote areas, thus contributing to its large-scale application and use. By including UK practitioners in the consultation and development stage, it was possible to assess potential geographical and cultural differences that could impact on the international appeal and relevance to the MT4C. No significant differences in terms of the barriers or enablers to change were identified by either the UK or the WA participant midwives.

## DISCUSSION

A variety of approaches have been used to create and develop implementation tools. These approaches can broadly be categorized as theory-driven, experience-driven, enhancement driven, participant or end user-driven, or a combination of any, or all four motivators.

Theory driven tools such as CFIR^9^ emerged through the use of systematic literature reviews. Following the review of almost 500 multi-disciplinary sources, Greenhalgh et al.^29^ developed a conceptual model to assist with the analysis of factors determining diffusion, dissemination, and implementation of innovations within healthcare. This conceptual model was later used as a starting point for the development of CFIR. Such frameworks and theories guide the change leader to develop a successful implementation strategy that considers and recognizes influential factors of the implementation process or explain why an implementation effort was not successful.

Created as a result of an evaluation and combination of pre-existing implementation theories and frameworks, CFIR^9^ represents a comprehensive typology of elements and factors that interact to explain and shed light on the implementation process. Unlike MT4C, CFIR is not a ‘tool’ that can be progressively worked through by the change leader. Instead, users are encouraged to evaluate each domain and then decide on the aspects relevant to their setting and develop ways they can be addressed. CFIR is undoubtedly widely used across multiple disciplines to help guide and inform implementation research^30-34^. However, anecdotal evidence would suggest the format may be overwhelming for a midwife new to change implementation, working in clinical practice or at the start of an implementation activity. It has also been argued that conceptual frameworks, such as CFIR may be of limited practical use when it comes to developing implementation strategies in practice^35^. The MT4C addresses the domains highlighted by CFIR as influential to successful implementation strategies. This provides sound theoretical support to the content of MT4C whilst simultaneously placing the domains within a midwifery specific context and in a format that is both user friendly and easily accessible.

Austin and Ciaassen^36^ similarly created a tool informed by the findings of a literature review. Designing their resource for use within social services organizations, they recognized the importance of evaluating readiness for change in advance of an implementation activity as a fundamental part of successful change implementation. Their findings highlighted the importance of organizational culture within implementation. Within the context of the development of MT4C, the issue of culture was discussed at both SAG meetings. Participants recognized that successful implementation was dependent on an organizational culture based upon mutual respect. The significance of culture was incorporated throughout the ‘Context’ domain within the MT4C with an additional note about the importance of culture and its potential to impact on implementation inserted into the tool preamble. The tool proposed by Austin and Ciassen^36^ comprises four key components extrapolated from their literature review, all are embedded in the elements which make up the three domains of MT4C. The MT4C considers the views, skills and experiences of all clinicians within the maternity care setting from the perspective of a midwife working within the clinical setting. Logistics and system requirements of the clinical midwifery setting in terms of the implementation of change in practice are also addressed.

Experience-driven theories and frameworks are derived as a result of researchers’ own experiences of the implementation process and, include factors and constructs found to be of significance within specific implementation activities. It was the personal experience of change implementation that enabled Barwick^37^ to create CARI, having reviewed and refined the work of Austin and Ciassen^36^; additional factors found to be significant to successful implementation within the authors own experience over ten years of evidence implementation were embedded into CARI. Initially designed for the context of behavioral health services in Canada, CARI has been deemed by Barwick^37^ as suitable for adaptation to other contexts, however, at the time of writing, a search of CINAHL and MEDLINE databases failed to show any published work in which such transformation has occurred allowing evaluation of the tool proposed flexibility.

Like CARI, MT4C was developed from a pre-existing readiness for change assessment tool, the UK NHS III Spread and Adoption tool. The components of CARI are similar to those used within MT4C, which is reassuring in terms of homogeneity with a current readiness for change assessment tool. However, the MT4C differs in that the changes were applied following consultation with change leader midwives from a range of clinical settings contributing to its broad-scale applicability. Unlike CARI, which is presented as checklists to be downloaded, completed, and then forwarded to the creators for interpretation, MT4C has been developed into a progressive web application which can be utilized on any hand-held or desktop device. The MT4C interface then creates an instant report which is emailed directly to the user. This streamlines the readiness for change assessment process removing the need for third-party involvement.

Kitson et al.^38^ made use of their collective experience within implementation to create PARIHS, a conceptual framework presented in the form of an equation. As a framework, PARIHS defines successful implementation as a result of the function of three fundamental factors, each having equal significance: evidence, context and facilitation. PARIHS is presented as a checklist of influencing factors used to map an implementation strategy. Still, as with many such frameworks, this approach relies upon a degree of knowledge and experience that potential change leaders new to implementation may be lacking. Kitson et al.^38^ acknowledged that PARIHS is a deductive framework and, at the time of its creation, had not been tested, calling upon others to make use of PARIHS to test its efficacy and validity as an implementation tool.

Seventeen years after developing the first version, Harvey and Kitson^39^ evaluated the efficacy of PARIHS by conducting a literature search of articles describing and assessing its use in practical implementation strategies. Through analysis of the feedback accessed through their literature review, Harvey and Kitson^39^ were able to determine that within PARIHS there was an inherent lack of focus on the individuals who were participating in the implementation strategy in addition to a lack of clarity in terms of defining the key concepts within the tool. This resulted in the PARIHS tool being deemed unwieldy and challenging for use by clinicians or those new to change implementation. Changes were made to PARIHS to address these perceived shortcomings. It was through this evaluation of others’ experiences of using their tool that Harvey and Kitson^39^ were able to refine the PARIHS framework and create the next iteration, which they named ‘i-PARIHS’.

In contrast to its original content, i-PARIHS suggests that successful implementation is a result of ‘facilitation of an innovation with the recipients in their (inner and outer) context’^39^. The constructs of Innovation and Recipient have been added to the framework and incorporate aspects such as the source of knowledge, novelty and trialability. These factors have been incorporated into the domains of the MT4C. In the revised i-PARIHS tool, the significance of the recipient is concerned not only with the interplay between the various teams and personnel involved with the implementation activity, but also the end benefactors of the innovation. The MT4C considers all members of the multi-disciplinary maternity care team in addition to addressing whether the proposed change is woman-centered, a fundamental criterion of midwifery practice.

The need to modify and adapt interventions during implementation strategies is addressed within the implementation tool created by Wiltsey Stirman et al.^40^. FRAME represents an extended Framework for Reporting Adaptations and Modifications to Evidence-based interventions. Wiltsey Stirman et al.^40^ acknowledge previous iterations of their tool did not place enough significance on aspects of modification and adaptation of the intervention which may be deemed essential and worthy of reporting when considering the overall success of an implementation strategy. FRAME was developed through the application of the findings of a literature review combined with the input of mental health providers. Data were consolidated by consensus to create a draft framework presented to stakeholders for further refinement. Consultation with stakeholders to ensure that the end tool was fit for purpose was a fundamental intention within the creation of the MT4C.

To create the Theoretical Domains Framework (TDF), Michie et al.^41^ consulted with three groups of experts: health psychology theorists, health service researchers, and health psychologists. Each group was used at specific stages of the development process according to the skills they inherently brought to the process. Thus, TDF was created through the evaluation of theory and its application to practice. Validation of TDF occurred through interviews of health psychologists from a range of settings to demonstrate broad-scale applicability of the fundamental theories used. Within the creation of the MT4C, a similar validation has occurred through consultation with change leader midwives from a range of clinical and geographical settings. The content of the domains determined by the participants has since been cross-referenced with CFIR, confirming that the pre-determined factors of successful implementation are incorporated.

Incorporating the opinions of target end-users may also improve the efficacy and utility of the final tool. Hull et al.^42^ created the Implementation Science Research Development (ImpRes) tool aimed at facilitating the implementation process for health researchers without prior training in implementation science. ImpRes was created through the combination of expert opinion and brainstorming, literature review, specialist input in the form of a review of domains and pilot testing both prospectively and retrospectively. The creation of ImpRes demonstrates the importance of both theory and practical application. This development approach is not dissimilar to that used for the creation of the MT4C.

Consultation with clinicians is fundamental to the overall success or failure of any implementation activity, as clinicians are at the forefront of patient care and the leaders in change. Timmings et al.^43^ recognize that ‘involving potential end-users in tool development is a critical step in ensuring the tool meets both functional goals … and usability needs’. Similarly, the creators of PARIHS and i-PARIHS recognize that the path to successful implementation should be mapped by participants ‘based on detailed analyses of their contextual and facilitative situation’^43^ and this notion of user involvement is represented in consideration of the needs, experience, or requirements of the target end-users. The input of these key stakeholders is directly sought during the development process of several tools and frameworks.

The value of clinician input was considered crucial for the development of MT4C and directly informed the decision to utilize PAR as the overarching methodology. This methodology provided practicing midwives an opportunity to openly discuss the challenges they have met within their implementation efforts and assist fellow and future change leaders through the development of a tool specifically designed to identify potential barriers and enablers to change. This will enable the implementation of evidence into practice to be more accessible for midwives regardless of their role or position within the larger institution of maternity healthcare. Furthermore, the MT4C is a direct result of consultation with end-users and not merely the product of academic theory application or personal experience alone.

Although initially guided by pre-existing work and tools in the same way that CFIR, CARI and FRAME were, the MT4C has been created with the specific input of change leader midwives who have direct experience of trying to implement a change of practice within the midwifery context. The MT4C is an enhancement of a pre-existing tool based on the input of target end-users, presented in the form of a progressive web application transcending many of the technical challenges inherent to the original tool, whilst meeting the unique requirements of the midwifery practice setting. The MT4C provides the user with a report outlining the identified barriers to change, to assist with the development of a suitable implementation strategy. By retrospectively applying the domains to recognized implementation framework it is possible to establish validity through the incorporation of recognized vital components of successful implementation frameworks. However, it also encompasses features that are unique to the midwifery setting.

Incorporating the views and experiences of target end-users ensures the functionality and usability of the MT4C meets the needs of midwives wanting to bring about change in the clinical setting. As frontline clinicians, it is unlikely that midwives will have specialist implementation science training or experience. Ensuring the final tool is user-friendly in terms of terminology, accessibility and functionality was a key aim of this study. The value of this is born out of the need for the creation of a supplementary guide to assist users of ImpRes^42^. Change leader midwives formulated the elements within each domain before deciding how the results are presented in the final report. This has ensured that the tool is not constructed from implementation science jargon and can be used by midwives regardless of prior knowledge or experience. The terminology used is broad enough to be applicable across a range of midwifery settings, yet specific enough to consider the peculiarities of contemporary maternity care environments.

### Limitations

A limitation of the study is that some barriers and enablers to midwifery practice change may not have been identified, however this will become clear in future testing of the MT4C. Additionally, participants were drawn from a range of settings, however there is a risk that the sample was not representative of the profession as participation was dependent on a process of self-selection. Finally, although the online version of the tool enabled midwives who were not able to attend the SAG in person to participate, the format did not allow for further discussion and clarification of points and issues raised. Therefore, the data obtained from the SAG were far richer than that generated via the online participation option.

## CONCLUSIONS

The aim of this study to develop a midwifery specific tool to identify barriers and enablers to evidence-informed practice change within the clinical setting, was achieved. The tool represents the first known attempt to develop such a tool. The MT4C provides midwives with a means of facilitating latest/best evidence-based improvements to maternity care. Including midwives from both Australia and the UK in the development of the MT4C has ensured the tool's fit for a broad range of practice settings internationally and offers potential for collaborative change efforts and for benchmarking. The necessity of the MT4C tool is assured through a review of the current literature. In addition to facilitating improvements in clinical care and professional autonomy, the MT4C identifies barriers to change in a range of settings. The identification of such obstacles will enable care providers to examine services and structures and make changes that could impact significantly on interdisciplinary working and collegial respect. The MT4C will illuminate the barriers to change and highlights the challenges midwives face in their attempts to bring about change to practice. Once these barriers have been identified, a discourse can be commenced to examine why they exist and how to remove them.

## Data Availability

The datasets used and/or analyzed during this study are available from the first author on reasonable request.
