# Supplementary material for: Development of a tool to identify barriers and enablers to practice innovation in midwifery: A participatory action research study
Source: Eur J Midwifery. 2023 Jan 30;7:1. doi: 10.18332/ejm/157459 (PMC9885374; doi:10.18332/ejm/157459)
Supplement: Supplementary file 1 [file EJM-7-1-s1.pdf]

Appendix 1

Elements removed from MT4C during round one SAG and online surveys

1. The Maternity team works within a culture of respect, trust, and open communication.
2. The proposed change is supported by consumers and midwives.
3. The evidence is relevant to my clinical area.
4. It will be straightforward to try the new practice in my area.
5. The change can be implemented within the current infrastructure.

© 2023 Davis S. D. et al.
